# Compassion as the reunion of feminine and masculine virtues in medicine

**Published:** 2017-10-07

**Authors:** Kiarash Aramesh

**Affiliations:** *Associate Professor, Medical Ethics and History of Medicine Research Center, Tehran University of Medical Sciences, Tehran, Iran; Scholar in Residence, Center for Healthcare Ethics, Duquesne University, Pittsburgh, PA, USA.*

**Keywords:** *Compassion*, *Virtue ethics*, *Futility*, *Doctor-patient relationship*, *End-of-life care*

## Abstract

The central role of the virtue of compassion in the shaping of the professional character of healthcare providers is a well-emphasized fact. On the other hand, the utmost obligation of physicians is to alleviate or eliminate human suffering. Traditionally, according to the Aristotelian understanding of virtues and virtue ethics, human virtues have been associated with masculinity. In recent decades, the founders of the ethics of care have introduced a set of virtues with feminine nature. This paper analyzes the notion of compassion as a common virtue between the traditional/masculine and care/feminine sets of virtues and shows that compassion is a reunion and merging point of both sets of human virtues. This role can be actualized through the development and promotion of compassion as an important part of the character of an ideal physician/healthcare provider. In addition, this paper argues that the notion of compassion can shed light on some important aspects of the contemporary debates on healthcare provider-patient relationship and medical futility. Despite the recent technological and scientific transformations in medicine, the interpersonal relationship between healthcare providers and patients still plays a vital role in pursuing the goals of healthcare. The virtue of compassion plays a central role in the establishment of a trust-based physician-patient relationship. This central role is discernible in the debate of medical futility in which making difficult decisions, depends largely on trust and rapport which are achievable by compassion in the physician and the recognition of this compassion by the patients and their surrogate decision makers.

## Introduction

Compassion has been called “the emotional and virtuous core of the desired professional attitude” in medicine (1). The Ethics Committee of *the American Society of Academic Emergency Medicine* (SAEM) considers compassion “a part of professional competence” which is “perhaps as important as technical competence” (1). These instances of emphasis on the central role of compassion in the shaping of the professional character of healthcare providers show the crucial importance of this concept in medicine and medical ethics.

Compassion is the internal/subjective reaction to the suffering of another sentient being(s) and is conjoined with recognition of the suffering, detestation and disapproval of that suffering, the feeling of personal responsibility for and engagement with the experience, and tendency to relieve the suffering with good intentions toward the sufferer(s) (2). Therefore, one can argue that compassion is always a good trait and there is not such a thing as “bad compassion”.

From the ancient times, the utmost obligation of physicians has been to alleviate or eliminate human suffering (3). Daniel Callahan calls this obligation “a foundation stone of the practice of medicine” (4). Patients’ suffering is not limited to pain or other symptoms of their diseases; it also encompasses their mental, social, and spiritual discomfort. This suffering originates from different sources including their disease, the treatment, their realistic or unrealistic fears and anxieties, their financial and social distresses, and other types of discomfort they experience through the course of their disease, treatment, and recovery (3). Alleviating patients’ suffering in a professional manner – not as a business – necessitates an ethical backbone that is composed of virtues such as compassion.

The broadness and existential importance of the concept of suffering and its importance in the life of a patient show how crucial the virtue of compassion is in the practice of medicine and in the pursuit of its goals (3). Suffering elicits the “impulse of compassion” in almost every normal human being (4), but it should develop into a virtue in physicians in order to render them more similar to the ideal physician.

This paper analyzes the notion of compassion as a common virtue between the traditional/masculine and care/feminine sets of virtues and shows that compassion as a reunion and merging point of both sets of human virtues has a crucial role in pursuing the goals of medicine and healthcare. This role can be actualized through the development and promotion of compassion as an important part of the character of an ideal physician/healthcare provider. In addition, this paper argues that the notion of compassion can shed light on some important aspects of the contemporary debates on healthcare provider-patient relationship and medical futility.

***Compassion as a virtue***

The main goal of medicine is to alleviate or eliminate suffering. Therefore, compassion is one of the most crucial virtues in pursuing the goals of healthcare and medicine. According to Alasdair MacIntyre, a virtue is a developed trait of a human’s character that tends to qualify him/her to realize the goals of a certain practice (in our case, medicine and health care) with excellence (1). Traditionally, according to the Aristotelian understanding of virtues and virtue ethics, human virtues have been associated with masculinity (5). This notion of ethical virtues had bestowed by dominance in the realm of philosophy for several centuries.

In the recent decades, however, the founders and advocates of the ethics of care have described and introduced a set of virtues with feminine nature (see below). This part of the paper portrays compassion as a common virtue between these two sets of universal virtues. Both the traditional and feminist theories of virtue ethics emphasize the importance of the virtue of compassion when it comes to healthcare ethics and healthcare provider-patient relationship.

At the end of this part, one can conclude that compassion as a virtue is the merging point of the masculine and feminine virtues in the realm of medicine and healthcare and is of crucial importance in shaping the character of a virtuous physician/healthcare provider.

***Compassion as a masculine virtue***

The traditional masculine virtue ethics is a moral theory mainly formulated by Aristotle. According to this moral theory, moral goodness and badness depends on the moral agent, and the development and establishment of moral virtues can empower a moral agent to act ethically in every practical situation.

Andre Comte-Sponville argues that contrary to sympathy, compassion is a virtue (6). Other scholars have also noticed the difference of concepts like sympathy, empathy, and pity with the concept of compassion (1). This differentiation, as described briefly below, shows how one can consider compassion as a virtue, not a feeling that can be morally good or bad.

Comte-Sponville differentiates sympathy and compassion in the following way. Sympathy, which means “fellow feeling”, is not a virtue by itself. Its goodness or badness depends on the “feeling” which is being shared between fellows. Having sympathy to malice intents is not good; therefore, sympathy, by itself, can never be a virtue. Compassion, however, is sympathy in suffering, and every form of suffering, even those originated from wrong causes such as wrongful jealousy or rivalry, deserves sympathy (6). In addition, compassion encompasses other specifics such as benevolence and an inclination to relieve the suffering (2).

Comte-Sponville brings Christ’s compassion for his executioners as an example of the goodness of compassion even for evil people who suffer because of their evil and malice acts, intents, and character (6). Andre Comte-Sponville argues that compassion, simultaneously, is a feeling and a virtue because we can feel it as a feeling and we can want and gain the capacity of being compassionate (6). In this sense, compassion is similar to love. In Buddhism, compassion is regarded as a great virtue. In Christianity and Islam, charity has the same status. However, charity is not mutually exclusive to compassion. As a matter of fact, the feeling and capacity of compassion can lead to and resemble charity (6).

As a virtue in its traditional/masculine sense, compassion is in close relation with biomedical and healthcare ethics. As mentioned above, the alleviation of suffering is a core obligation/goal in medicine and healthcare. The virtue which targets suffering is compassion. Therefore, the pursuit and realization of this main goal of medicine depends on the establishment of this virtue in healthcare providers/physicians.

***Feminine virtues vs. masculine ethics***

The ethics of care is among the most recently emerged moral theories and has attracted a large deal of attention in the recent decades (7). Its new and innovative approach and viewpoint in dealing with ethical issues has shed light on some formerly dark and overlooked parts of human beings’ moral obligations and duties. 

As a moral theory, ethics of care has implications and influences on biomedical ethics. Healthcare is one of the most prominent manifestations of care and caring relation in the world of humanity (8). Therefore, it is evident that ethics of care has much to say when it comes to health, healthcare, and the goals of medicine.

The ethics of care, as a distinct moral theory, was born inside the feminist ethics. The founders of this theory were feminist philosophers who found the caring nature of femininity of enormous ethical value (9). As described above, traditionally, in moral theories and even in moral psychology, it was taken for granted that ethical virtues are stronger in males (5). This was because ethical norms and virtues like justice and impartiality were consistent with the role of males as hunters and breadwinners. The role of females, as caretakers, had almost always been underestimated, morally speaking. According to the founders of the ethics of care, however, this caring nature of the female role is of utmost moral superiority and importance and can be considered as a basis for a self-sufficient moral theory. Although some of them believe that the ethics of care is not a category of virtue ethics, still it is clear that this moral theory is founded based on considering care and compassion as unambiguous virtues. 

One of the main themes of the ethics of care is considering partiality as a virtue. In the traditional/masculine virtue ethics, justice and impartiality have always been indubitable virtues. However, in the ethics of care, special caring relations accompany the obligation of special care and partiality. This is true in the case of physician-patient relationships.

***Compassion as a feminine virtue***

Medical ethics and professionalism ask physicians to always prioritize the health and health needs of their own patients. This priority stems from a relationship, the physician/doctor/healthcare provider-patient relationship. It seems that the typical model of the ethics of care shows itself in this case. The physician should be partial and prioritize his/her patients because of the specific relationship established between them. 

This partiality is demonstrated in the forms of care and compassion. Therefore, one can argue that compassion is a virtue that has a central role in feminine virtue ethics or ethics of care. The virtues of care and compassion are aimed at the realization of the goals of medicine as described below.

**Compassion and the goals of healthcare**

Daniele Callahan specifies the goals of medicine as the prevention of disease and injury and the promotion and safeguarding of health, relief from all kinds of suffering resulted by maladies, the treatment of disease and provision of care for non-curable diseases, and the evasion of a premature death and the pursuit of a serene death (10). It seems that the virtue of compassion plays crucial roles in the realization of all of these four goals of medicine/healthcare. In this part of this paper, the role of compassion in realizing these goals is described under two major topics of healthcare provider-patient relationship and medical futility.

***Compassion and healthcare provider-patient relationship***

The root of the word “compassion” is in Latin where it means “suffering with”, equal to the Greek root of the word “sympathy” (2). It is interesting that compassion shares its Latin root with the word “patient” meaning sufferer (6). This common root symbolically shows the relation between compassion and caring for parents and relieving their suffering as embodied in healthcare and medicine.

Medicine relies on science, but is not merely a sort of science. It is also an art; the art of establishing a healing and trust-based relationship with the patients. This art depends on certain virtues in physicians, among which is the crucial one of compassion.

Recent developments in medical technologies along with reliance on science have transformed the doctor-patient relationship in the post-World War II era (3). However, these changes and evolutions have not led to the elimination of humanistic aspects of the doctor-patient relationship and its transformation into a mechanical/machine-like relationship. Therefore, the therapeutic relation still depends largely on development of trust and rapport between physician and patient. This trust and rapport are achieved when patients realize that their doctor recognizes their suffering, feels for them, and intends to alleviate or eliminate their suffering. This is the definition of compassion as previously described in this paper. Therefore, it seems that despite all the evolutions and transformations in modern medicine, the cornerstone of the doctor-patient relationship is still personal virtues, and among them, the important one of compassion.

***Compassion and the notion of medical futility***

Medical futility refers to that sort of medical interventions that do not have any benefit for the patient, and therefore, should be forsaken (11). Medical futility encompasses a wide range of routine and common instances of medical care such as prescribing antibiotics for the common cold to some sensitive and emotional instances like withdrawing a life sustaining treatment at the end of life (12). 

Even if the patients or their families ask, the healthcare providers have no obligation to provide a futile treatment. Instead, they are ethically obliged to refuse to provide any futile interventions (11). Determining which intervention is futile for a given patient, however, is not always easy and straightforward and cannot be decided upon by only the physicians (12). As a matter of fact, both the technical/scientific knowledge of the physician/healthcare provider and the perception of the patient/surrogate decision maker(s) of his/her suffering are important in making an ethical decision. 

The virtue of compassion is crucial in optimizing the role of the physician in such a situation. The physician’s sympathetic and compassionate understanding of the patient’s suffering is the cornerstone of a constructive dialogue which leads all the involved parties through the difficult course of decision making on medical futility, especially in sensitive and emotional cases such as those related to end-of-life care and withholding or withdrawing life-sustaining interventions.

It seems that compassion, as a virtue can be helpful in decision making regarding medical futility through the following aspects:

1. Compassion helps the physician to understand the needs, fears, urges, and desperations behind the decisions of the patient or the surrogate decision-maker.
2. Compassion, when realized and recognized by the patient and his/her surrogate decision-maker, helps to establish a relationship based on trust between two parties and facilitates dialogue, common decision-making, and the obtaining of a consensus. Because in this case, the patient and his/her relatives feel that the decisions and suggestions of the physician are in the best interest of the patient. Otherwise, they might doubt that there may be financial or other unrelated goals behind the suggestions of the physician that is very destructive to trust and relationship.

## Conclusion

Taking a look at the history of debates in contemporary biomedical ethics clearly shows that no single moral theory has a monopoly on the realm of truth in biomedical and healthcare ethics. Instead, in each situation and in analyzing each specific issue or searching for each specific ethical solution, one or more of ethical theories are shown to be helpful and reliable. In this paper, the viewpoint of virtue ethics has been used and analyzed to shed light on some aspects of biomedical ethics and to show that compassion as a virtue plays a vital role in the pursuit of the goals of healthcare and medicine.

Healthcare, in practice, involves a great deal of interpersonal involvements and interactions. Therefore, the character of the physician/healthcare provider is of crucial importance. This importance paves the way for virtue ethics to play a considerable role in analyzing and problem-solving in healthcare provider-patient issues. In this context, compassion, as a virtue, is of importance in pursuing the goals of medicine/healthcare as described above.

Compassion is a common virtue between the traditional/masculine and feminist/feminine theories of virtue ethics and, according to both of them, is a crucial virtue in biomedical ethics and medical professionalism. The traditional/masculine sense of the virtue of compassion can strengthen the relationship between physician and patient with trust and mutual understanding. In addition, the partiality resulted from this relationship shows itself in the form of prioritizing one’s own patients and is compatible with the feminine account of virtues such as compassion and care. 

Despite the recent technological and scientific transformations in medicine, the interpersonal relationship between healthcare providers and patients still plays a vital role in pursuing the goals of healthcare. The virtue of compassion plays a central role in the establishment of an effective and trust-based physician-patient relationship. This central role is discernible in the debate of medical futility in which making difficult decisions, especially in emotional situations such as end-of-life care, depends largely on trust and rapport which are achievable by virtue of compassion in the physician and the recognition of this compassion by the patient and his/her surrogate decision makers.

Finally, compassion can be called the merging and reunion point of the feminine and masculine virtues in pursuing the goals of healthcare and medicine. Developing and strengthening this virtue should be a part of both formal and hidden curriculums in medical education in every medical school all over the world.
